# Kinematic alignment versus mechanical alignment in primary total knee arthroplasty: an updated meta-analysis of randomized controlled trials

**DOI:** 10.1186/s13018-022-03097-2

**Published:** 2022-04-04

**Authors:** Binfeng Liu, Chengyao Feng, Chao Tu

**Affiliations:** 1 Department of Orthopaedics, The Second Xiangya Hospital, Central South University, Changsha, 410011 Hunan China; 2 Zhengzhou University People’s Hospital, Henan Provincial People’s Hospital, Henan, Zhengzhou, 450003 China; 3 Hunan Key Laboratory of Tumor Models and Individualized Medicine, The Second Xiangya Hospital of Central South University, Changsha, 410011 Hunan China

**Keywords:** Kinematic alignment, Mechanical alignment, Total knee arthroplasty, Total knee replacement, Meta-analysis

## Abstract

**Background:**

The purpose of this study was to perform an updated meta-analysis to compare the outcomes of kinematic alignment (KA) and mechanical alignment (MA) in patients undergoing total knee arthroplasty.

**Methods:**

PubMed, EMBASE, Web of Science, Google Scholar, and the Cochrane Library were systematically searched. Eligible randomized controlled trials regarding the clinical outcomes of patients undergoing total knee arthroplasty with KA and MA were included for the analysis.

**Results:**

A total of 1112 participants were included in this study, including 559 participants with KA and 553 patients with MA. This study revealed that the Western Ontario and McMaster Universities Osteoarthritis Index, Knee Society Score (knee and combined), and knee flexion range were better in the patients with kinematic alignment than in the mechanical alignment. In terms of radiological results, the femoral knee angle, mechanical medial proximal tibial angle, and joint line orientation angle were significantly different between the two techniques. Perioperatively, the walk distance before discharge was longer in the KA group than in the MA group. In contrast, other functional outcomes, radiological results, perioperative outcomes, and postoperative complication rates were similar in both the kinematic and mechanical alignment groups.

**Conclusions:**

The KA technique achieved better functional outcomes than the mechanical technique in terms of KSS (knee and combined), WOMAC scores, and knee flexion range.

*PROSPERO trial registration number* CRD42021264519. Date registration: July 28, 2021.

**Supplementary Information:**

The online version contains supplementary material available at 10.1186/s13018-022-03097-2.

## Background

Knee osteoarthritis (OA) is one of the most common degenerative joint diseases that impose a substantial socioeconomic burden on society and health care systems [1]. The incidence of knee OA has significantly increased in recent decades due to the continuous increase in obesity and the aging population in the world [2]. Total knee arthroplasty (TKA) is the most effective treatment for end-stage knee OA, which can significantly alleviate pain and improve quality of life. Meanwhile, new technologies have further improved the clinical efficacy and safety of TKA, including novel concept implants, novel extramedullary guides, and computer-assisted surgery [3–5]. Cristian Aletto et al. revealed that computer-assisted TKA ensures good functional outcomes [3]. As a result, the number of patients undergoing TKA has steadily increased each year as these medical technologies continue to advance [6]. Previous studies claim that, by 2030, 3.8 million people will have undergone TKA each year [7]. The accurate restoration of knee alignment is essential to the success of TKA, which is vital for the recovery of the patient's postoperative function and implant survival [8]. Currently, the alignment methods of the lower limbs used in TKA mainly include kinematic alignment (KA) and mechanical alignment (MA).

MA is the traditional alignment method in TKA and has been used for more than 30 years. MA aims to create a neutral hip–knee–ankle angle (HKA) to restore the overall limb alignment to a neutral position [9]. From a mechanical perspective, MA can optimize load distribution in patients undergoing TKA and prolong prosthesis survival by reducing polyethylene wear and component loosening [10]. Previous studies have also reported that the MA technique can improve patient satisfaction and relieve pain [11]. For instance, navigation-assisted TKA can effectively replicate the neutral MA of the knee, thereby reducing alignment outliers [12]. However, it was reported that up to 25% of patients undergoing MA in TKA still have unsatisfactory outcomes [13, 14]. This may be due to abnormal touch kinematics caused by MA changing the limb axis of the knee, thus resulting in substandard patient satisfaction [15].

In contrast, the KA technique aims to restore the alignment and kinematics of the TKA implant, thus ensuring its match to the pre-osteoarthritis anatomy. Due to the disadvantages of MA, the clinical application of KA in TKA has become increasingly popular since Howell et al. introduced it in 2006 [16]. The KA technique was the preferential method to place the knee implant in a natural anatomical position, compensate for the tibia and femur rotation changes, and preserve the original soft-tissue envelope. It reduces the loosening of soft tissues and ligaments around the knee and achieves better physiological kinematics of the knee [17, 18]. To date, accumulating evidence has demonstrated that KA in TKA will also help patients achieve better functional outcomes and alleviate postoperative pain [13, 19, 20]. However, several limitations remain in this technique: Restoring natural varus can increase the contact stress between the tibiofemoral and patellofemoral joints, which may lead to an increased risk of early implant dysfunction and failure.

Currently, no systematic evidence exists regarding whether the KA technique can attain similar or greater clinical outcomes than the classical MA technique in TKA. Although several randomized control trials (RCTs) and meta-analyses compared the clinical outcomes of KA and MA in TKA, the results remain controversial. For instance, Gao et al*.* [21] reported that patients undergoing KA in TKA had better clinical outcomes than patients undergoing MA in TKA. In contrast, another study revealed that KA and MA achieved similar results in TKA [22]. Furthermore, there have been several new RCTs in recent years, which have not been included in previous meta-analyses. Therefore, an updated meta-analysis is necessary to further explore whether KA is superior to MA. Accordingly, the aim of the current study was to conduct an updated meta-analysis of RCTs to evaluate the clinical differences, including the functional, radiological, perioperative, and complication results between the KA technique and the traditional MA technique in patients undergoing TKA.

## Methods

### Literature search strategy

In compliance with the referenced guidelines [23], two independent reviewers conducted a systematic search for relevant studies in PubMed, EMBASE, Web of Science, Google Scholar, and the Cochrane Library (from inception to January 17, 2022). The search terms consisted of Kinematic, Kinematical, Kinematically, Kinematic alignment, KA, Mechanical, Mechanically, Mechanical alignment, MA, osteoarthritis, OA, total knee replacement, total knee arthroplasty, TKA, and TKR. The language was limited to English. In addition, to identify other relevant potential research, the references of retrieved studies and previous relevant meta-analyses were further screened. This meta-analysis has been registered with the International Prospective Register of Systematic Reviews (PROSPERO; CRD42021264519).

### Inclusion criteria and exclusion criteria

Studies were included in this study if they met the following criteria: (1) all RCTs compared KA with the MA technique in TKA; (2) the participant underwent primary TKA using the KA or MA technique; (3) the experimental and control groups were KA and MA, respectively; and (4) outcome indices included the knee functional score, postoperative radiological results, perioperative results, and complications. The exclusion criteria were as follows: (1) review articles, case series or case reports, retrospective studies, letters, nonhuman studies, and cadaver studies; (2) research published in languages other than English; and (3) studies that lacked comparative data.

After excluding duplicate publications, two investigators (BFL and CYF) selected studies independently as per the above criteria. First, initial eligibility was screened by the titles and abstracts of all identified studies. Subsequently, the full text of all potentially eligible studies was reviewed. Disagreements were resolved through discussion between the two investigators, and any disputes were resolved by a third investigator (CT) to reach a consensus.

### Data extraction

Two independent reviewers extracted data (BFL and CYF). A third reviewer (CT) was an assistant to resolve any disagreements by discussion and consensus. The following characteristics were extracted from individual eligible studies: name of the first author, publication years, country, study type, sample size, average age, follow-up times, and outcomes.

The outcomes included functional, radiological, perioperative, and complication results. Primary outcomes included functional and radiological results, while secondary outcomes included perioperative and complication outcomes. Of them, the functional outcomes comprised the Knee Society Score (KSS), Knee Injury and Osteoarthritis Outcome Score (KOOS), Western Ontario and McMaster Universities Osteoarthritis Index (WOMAC), Oxford Knee Score (OKS), EuroQoL 5-dimension questionnaire (EQ-5D), Forgotten Joint Score (FJS), and range of motion (ROM). The radiological results consisted of the HKA, femoral knee angle (FKA), mechanical medial proximal tibial angle (mMPTA), mechanical lateral distal femoral angle (mLDFA), joint line orientation angle (JLOA), tibial slope (TS), and femoral flexion–extension angle (FFA). Perioperative outcomes included operative time (OT), change in hemoglobin (CHb), wound length (WL), walking distance (WD), and hospital stay (HS). The complication results were divided into two subgroups: major complications and minor complications. The major complications included any complications that resulted in reoperation or revision, such as deep infection, patellar dislocation, and implant loosening, while minor complications referred to those that would not lead to deep infections or require revision, including postoperative pain, swelling, stiffness, and recurrent hemarthrosis.

### Risk-of-bias assessment

Two independent reviewers (BFL and CYF) evaluated the methodological quality of the enrolled studies by using the Cochrane Collaboration's tool. This tool focuses on the trial's internal validity and assessment of the risk of possible bias in different phases of the trial. In addition, a funnel plot was used to assess publication bias. Disagreements were resolved through discussion.

### Statistical analysis

Review Manager (RevMan 5.3; The Nordic Cochrane Centre, The Cochrane Collaboration, Copenhagen, Denmark) was used to conduct relevant analysis in this study. In terms of continuous data, the mean difference (MD) and 95% confidence interval (CI) were applied for data analysis. The Mantel–Haenszel odds ratio (OR) and 95% CI will be used as effect measurements for categorical data. Heterogeneity was assessed using the Cochrane *Q* test (Chi-square test), and *p* < 0.1 was set as the level of significant heterogeneity. *I*^2^ was also used for the quantitative analysis of heterogeneity. *I*^2^ < 50% indicates no heterogeneity, and the fixed-effect model was used. *I*^2^ > 50% was considered too strong heterogeneity, and a random-effects model was adopted. If necessary, sensitivity analysis was conducted by omitting individual studies consecutively to evaluate their impact on this study. The overall effect was evaluated by the *Z* test, and *p* < 0.05 was considered to indicate statistical significance.

## Results

### Characteristics of the included studies

**Fig. 1:**
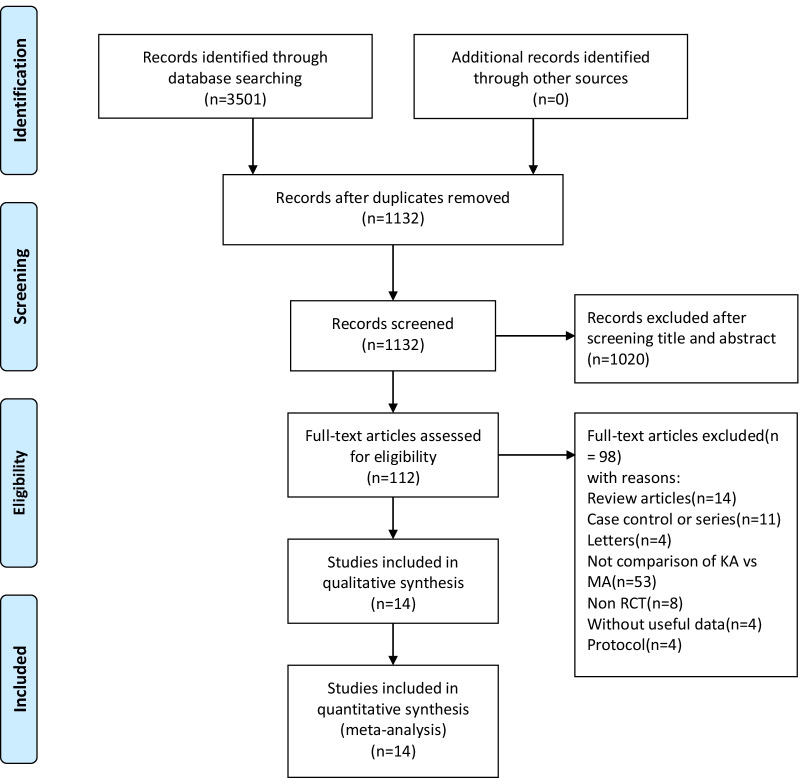
The search process and the screening of the articles for identifying the eligible studies

Details of the literature search and study selection are shown in Fig. 1. According to the search strategy, and the inclusion and exclusion criteria, fourteen RCTs were identified and included in this updated meta-analysis. The fourteen RCTs [19, 20, 24–35] were all reported in English, and the total sample size of the included study was 1112 cases, with 559 cases and 553 cases in the KA or MA technique, respectively. Table 1 summarizes these characteristics.

**Table 1:** Characteristics of the studies included

References	Years	Location	Study design	Sample size		Mean age		Female		Follow-up times (months)	Outcomes
				KA	MA	KA	MA	KA	MA		
Kaneda [35]	2021	Japan	RCT	8	2	76.3	75	NA	5	12	HKA, mLDFA, mMPTA,FFA,TS
Matsumoto [29]	2020	Japan	RCT	30	30	74.2	75.5	25	26	12	HKA, mLDFA, mMPTA, KSS, ROM
Young [25]	2020	New Zealand	RCT	47	48	NA	NA	NA	NA	60	KSS, WOMAC, OKS, EQ-5D, FJ, Complication
MacDessi [31]	2020	Australia	RCT	70	68	67.4	69.0	40	34	12	HKA, mLDFA, mMPTA, KOOS, EQ-5D, FJS, OT
McEwen [28]	2020	New Zealand	RCT	41	41	NA	NA	NA	NA	24	HKA, mLDFA, mMPTA, JLOA, FFA,TS, ROM, KOOS, OKS, FJS, Complication
Laende [32]	2019	Canada	RCT	24	23	64	63	16	17	24	HKA, mMPTA, OKS
Yeo [26]	2018	South Korea	RCT	30	30	72	74	25	27	96	HKA, mLDFA, mMPTA, FFA, TS,WOMAC, ROM
Matsumoto [30]	2017	Japan	RCT	30	30	75.3	76.1	18	20	24	HKA, JLOA, KSS, ROM
Waterson [27]	2016	United Kingdom	RCT	36	35	NA	NA	NA	NA	12	KSS, ROM, EQ-5D
Calliess [33]	2016	Germany	RCT	100	100	67	70	61	57	12	HKA, mLDFA, mMPTA, FFA, TS, WOMAC, Complication
Young [24]	2016	New Zealand	RCT	49	50	72	70	24	24	24	HKA, FKA, mLDFA, mMPTA, TS, KSS, WOMAC, ROM, OKS, EQ-5D, FJS, OT, WL, HS, Complication
Belvedere [34]	2015	Italy	RCT	6	11	NA	NA	NA	NA	6	KSS
Dossett [20]	2014	United States	RCT	44	44	66	66	3	6	24	HKA, FKA, mLDFA, mMPTA, JLOA, KSS, WOMAC, ROM, OKS, HS,CHb,WD, Complication
Dossett [19]	2012	United States	RCT	41	41	65	66	2	6	6	HKA, FKA, mLDFA, mMPTA, JLOA, FFA, TS, KSS, WOMAC, ROM, OKS, Complication, OT, WL, HS, CHb, WD

*KA* kinematic alignment, *MA* mechanical alignment, *HKA* hip–knee–ankle angle, *FKA* femoral knee angle, *mLDFA* mechanical lateral distal femoral angle, *mMPTA* mechanical medial proximal tibial angle, *JLOA* joint line orientation angle, *FFA* femoral flexion–extension angle, *TS* tibial slope, *KSS* knee society score, *KOOS* Knee Injury and Osteoarthritis Outcome Score, *WOMAC* Western Ontario and McMaster Universities Osteoarthritis Index, *OKS* Oxford Knee Score, *EQ-5D* EuroQoL 5-dimension questionnaire, *FJS* Forgotten Joint Score, *ROM* range of motion, *OT* operative time, *WL* wound length, *HS* hospital stay, *CHb* change in hemoglobin, *WD* walking distance, *NA* not applicable, *RCT* randomized controlled clinical trials

### Risk of bias and publication bias

**Fig. 2:**
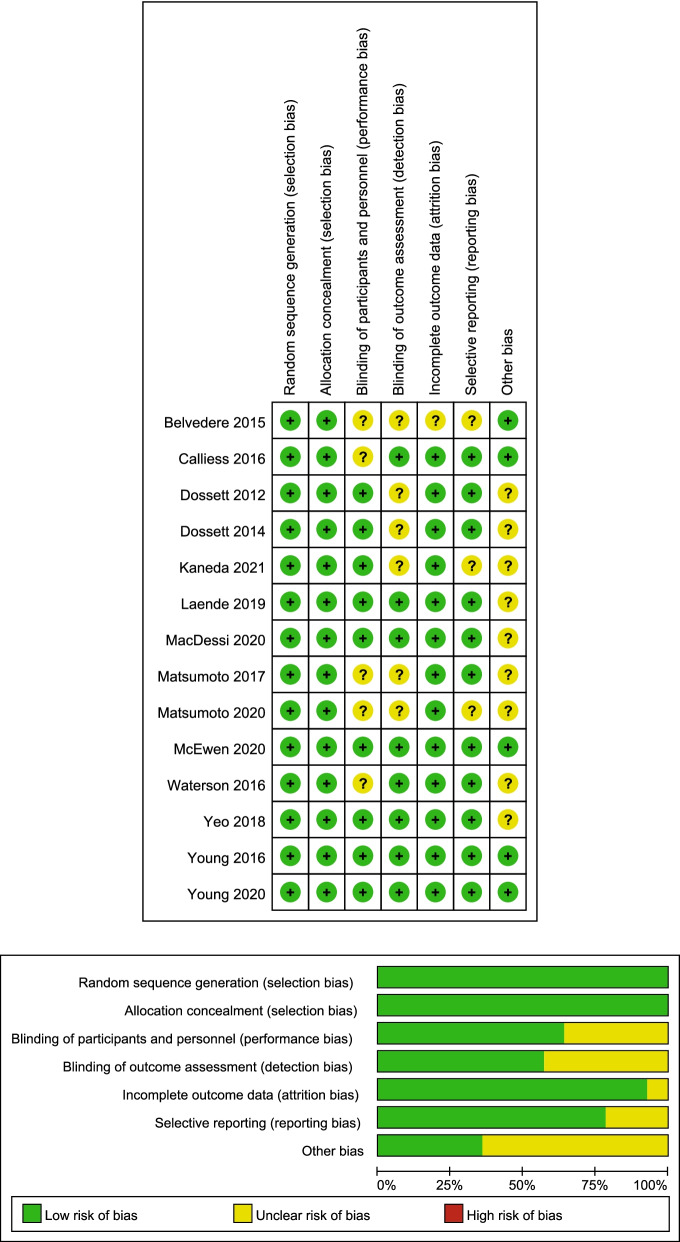
Risk-of-Bias Assessment summary

Overall, all included RCTs were evaluated for risk bias according to the seven aspects as follows: random sequence generation, allocation concealment, blinding of participants and personnel, blinding of outcome assessment, incomplete outcome data, selective reporting, and other bias. The results showed that all the RCTs were considered at low risk of bias. The details of the risk-of-bias assessment results are presented in Fig. 2. Publication bias was measured by a funnel plot. As demonstrated in Additional file 1: Fig. S1, low publication bias was indicated as the funnel plot showed symmetry.

### Functional results

**Fig. 3:**
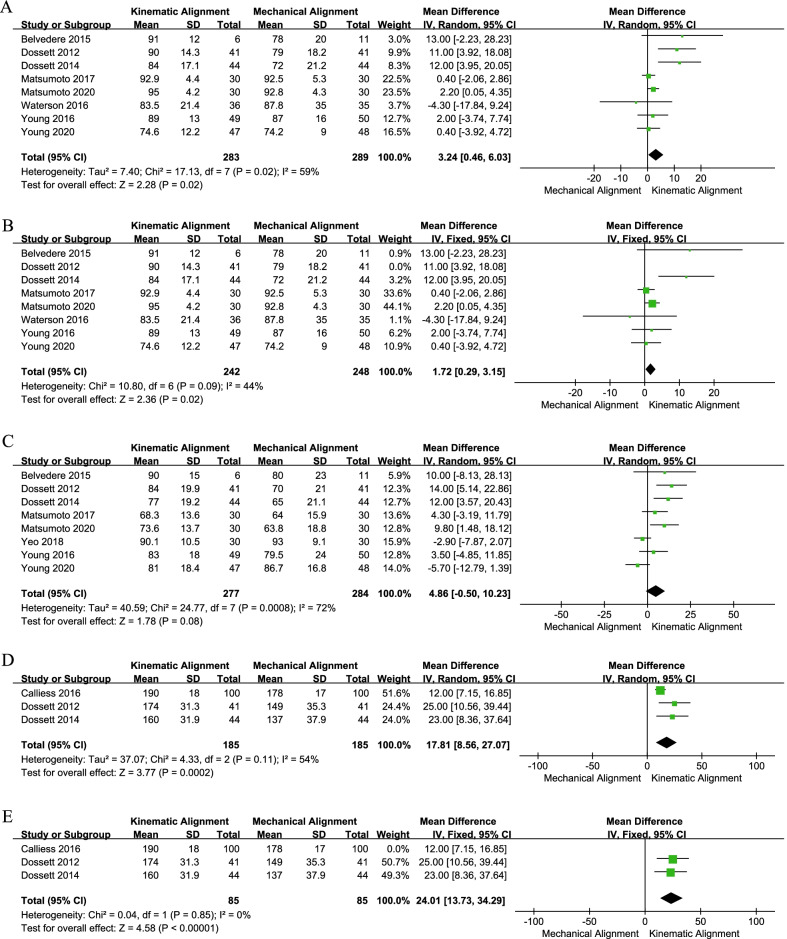
Forest plot of KSS between kinematic alignment and mechanical alignment in total knee arthroplasty. **a** KSS (knee), **b** the sensitivity analysis results of KSS (knee), **c** KSS (function), **d** KSS (combined), **e** the sensitivity analysis results of KSS (combined). *KSS* knee society score, *CI* confidence interval, *IV* inverse variance

As shown in Fig. 3a, eight studies with 572 patients were included in the KSS (knee) evaluation [19, 20, 24, 25, 27, 29, 30, 34]. The pooled MD was 3.24 (95% CI 0.46–6.03), and I^2^ was 59%. After dropping the research performed by Dosset et al. [19], the *I*^2^ dropped to 44%, and the pooled MD (1.72, 95% CI 0.29–3.15) was consistent with previous analysis (Fig. 3b). Three studies, including a total of 370 participants, assessed the KSS (combined) with pooled data (MD = 17.81, 95% CI 8.56–27.07, *I*^2^ = 54%, Fig. 3d) [19, 20, 33]. The sensitivity analysis results indicated that the heterogeneity mainly came from Calliess's studies [33] (Fig. 3e). The above results indicated that the KA group had better KSS (knee) and KSS (combined) scores than the MA group. However, we also discovered no significant differences in KSS (function) between the KA and MA groups (MD = 4.86, 95% CI − 0.50 to 10.23, *I*^2^ = 72%, Fig. 3c) [19, 20, 24–26, 29, 30, 34]. After gradually removing these eight studies, we found no significant change in heterogeneity, indicating that the pooled analysis results of KSS (function) were stable (Table 2).

**Table 2:** The sensitivity analysis results of KSS (function)

Study excluded	Remaining samples (KA/MA)	Overall effect			Heterogeneity	
		MD	95% CI	*p* value	*I*^2^ (%)	*p* value
Belvedere [34]	271/273	4.56	− 1.09 to 10.21	0.11	75	0.0005
Dossett [19]	236/243	3.47	− 1.80 to 8.74	0.20	67	0.006
Dossett [20]	233/240	3.78	− 1.77 to 9.32	0.18	70	0.003
Matsumoto [30]	247/254	5.07	− 1.20 to 11.33	0.11	76	0.0004
Matsumoto [29]	247/254	4.16	− 1.66 to 9.98	0.16	73	0.001
Yeo [26]	247/254	6.30	0.71 to 11.89	0.03	65	0.009
Young [24]	228/234	5.17	− 1.01 to 11.34	0.10	76	0.0004
Young [25]	230/236	6.49	1.08 to 11.89	0.02	67	0.006

*MD* mean difference

**Fig. 4:**
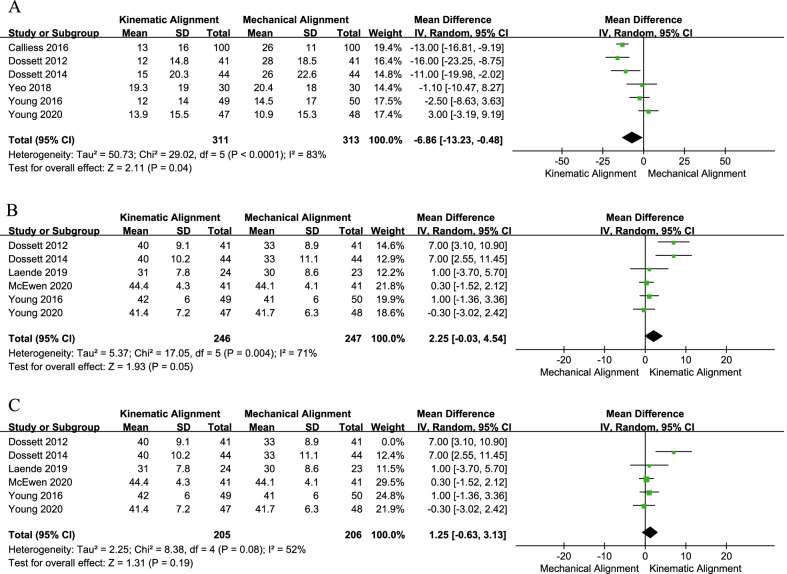
Forest plot of WOMAC and OKS between kinematic alignment and mechanical alignment in total knee arthroplasty. **a** WOMAC, **b** OKS, **c** the sensitivity analysis results of OKS. *WOMAC* Western Ontario and McMaster Universities Osteoarthritis Index, *OKS* Oxford Knee Score

**Fig. 5:**
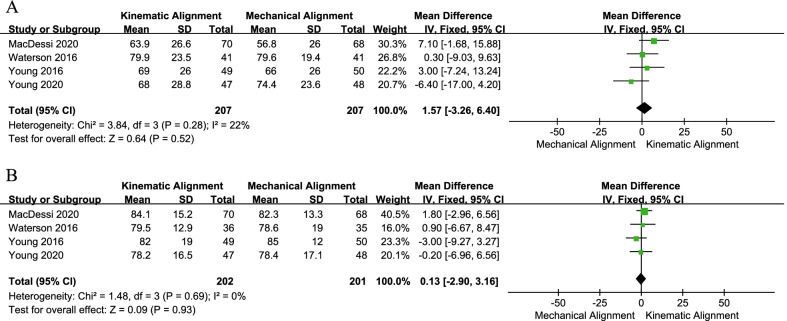
Forest plot of FJS and EQ-5D between kinematic alignment and mechanical alignment in total knee arthroplasty. **a** FJS, **b** EQ-5D. *FJS* Forgotten Joint Score, *EQ-5D* EuroQoL 5-dimension questionnaire

Six studies compared the WOMAC score [19, 20, 24–26, 33] and the OKS score [19, 20, 24, 25, 28, 32]. The pooled results (Fig. 4a) suggested that the WOMAC score in the KA technique group was better than that in the MA technique group (MD = − 6.86, 95% CI − 13.23 to − 0.48, *I*^2^ = 83%). Meanwhile, the sensitivity analysis result suggested that the analysis result was robust (Table 3). However, the pooled results of OKS showed a similar mean score between the two groups (MD = 2.25, 95% CI − 0.03 to 4.54, *I*^2^ = 71%, Fig. 4b). When excluding the study conducted by Dosset et al. [19], the pooled analysis result did not change (MD = 1.25, 95% CI − 0.63 to 3.13, *I*^2^ = 52%). In addition, all four RCTs [24, 25, 28, 31] reported FJS, and the analyzed results (MD = 1.57, 95% CI − 3.26 to 6.40, *I*^2^ = 22%, Fig. 5a) indicated no significant difference in FJS between the two techniques.

**Table 3:** The sensitivity analysis results of WOMAC

Study excluded	Remaining samples (KA/MA)	Overall effect			Heterogeneity	
		MD	95% CI	*p* value	*I*^2^ (%)	*p* value
Calliess [33]	211/213	− 5.37	− 12.43 to 1.69	0.14	78	0.001
Dossett [19]	270/272	− 5.05	− 12.06 to 1.95	0.16	83	< 0.0001
Dossett [20]	267/269	− 6.11	− 13.47 to 1.25	0.10	86	< 0.00001
Yeo [26]	281/283	− 7.83	− 14.89 to − 0.76	0.03	85	< 0.0001
Young [24]	262/263	− 7.76	− 15.21 to − 0.31	0.04	84	< 0.0001
Young [25]	264/265	− 9.06	− 14.69 to − 3.42	0.002	72	0.007

*MD* mean difference

**Fig. 6:**
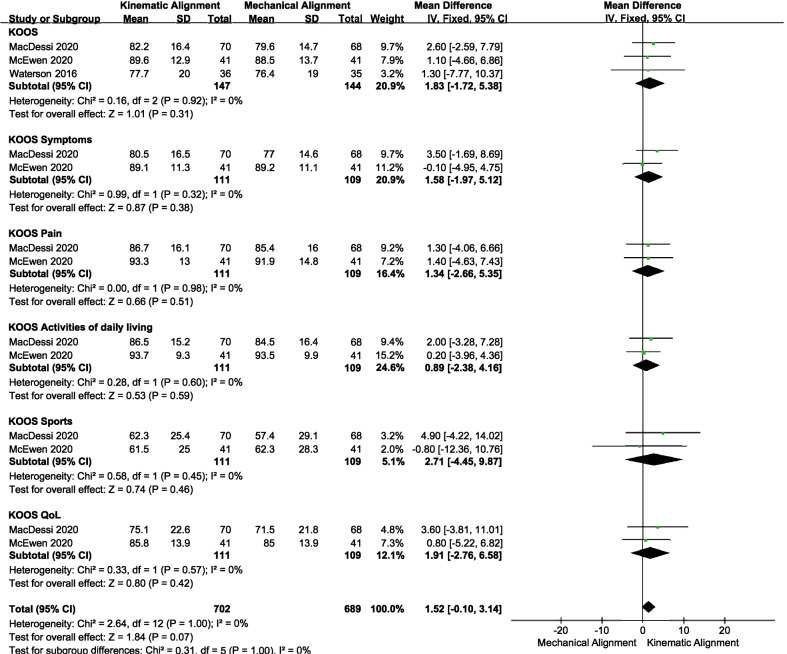
Forest plot of KOOS between kinematic alignment and mechanical alignment in total knee arthroplasty. *KOOS* Knee Injury and Osteoarthritis Outcome Sore

Four studies [24, 25, 27, 31] reported EQ-5D data, and the results revealed no significant difference between the two groups (MD = 0.13, 95% CI − 2.90 to 3.16, *I*^2^ = 0%, Fig. 5b). Similarly, three articles [27, 28, 31] assessed the KOOS. The pooled results indicated no significant difference in KOOS, KOOS pain, KOOS symptoms, KOOS activities of daily living, KOOS sports, or KOOS QoL between KA and MA (Fig. 6).

**Fig. 7:**
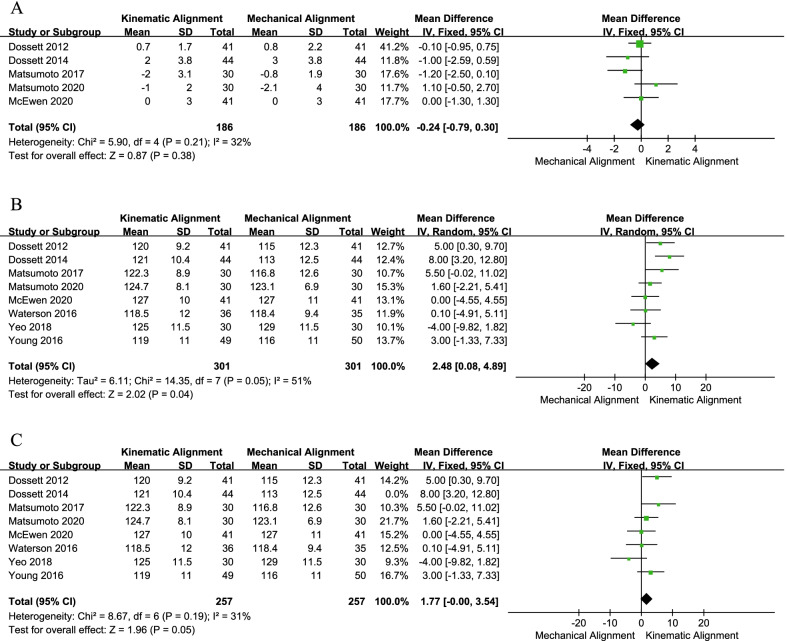
Forest plot of ROM between kinematic alignment and mechanical alignment in total knee arthroplasty. **a** ROM (extension), **b** ROM (flexion), **c** the sensitivity analysis results of ROM (flexion). *ROM* range of motion

A total of eight RCTs, including five trials [19, 20, 28–30], compared the extension range, angle and eight studies [19, 20, 24, 26–30] compared the flexion range angle. For the extension range angle, the pooled results indicated no significance between the two approaches (MD = − 0.24, 95% CI − 0.79 to 0.30, *I*^2^ = 32%, Fig. 7a). However, the pooled MD in flexion ROM was 2.48 (MD = 2.48, 95% CI 0.08–4.89, *I*^2^ = 51%, Fig. 7b), which means the KA technique had a higher ROM of flexion than the MA. Moreover, the results of the sensitivity analysis also support this finding (MD = 1.77, 95% CI − 0.00 to 3.54, *I*^2^ = 31%, Fig. 7c).

### Radiological results

**Fig. 8:**
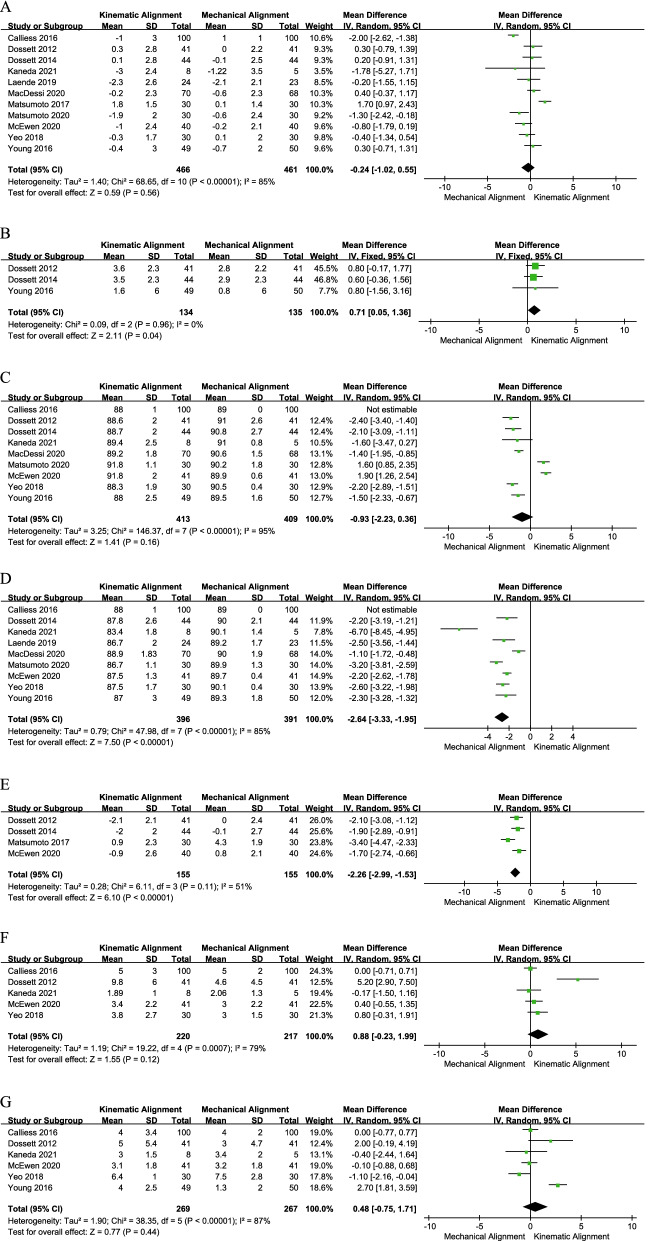
Forest plot of radiological results between kinematic alignment and mechanical alignment in total knee arthroplasty. **a** HKA, **b** FKA, **c** mLDFA, **d** mMPTA, **e** JLOA, **f** FFA, **g** TS. *HKA* hip–knee–ankle angle, *FKA* femoral knee angle, *mLDFA* mechanical lateral distal femoral angle, *mMPTA* mechanical medial proximal tibial angle, *JLOA* joint line orientation angle, *FFA* femoral flexion–extension angle, *TS* tibial slope

The pooled result of HKA [19, 20, 24, 26, 28–33, 35] indicated that the two techniques have a similar HKA (MD = − 0.24, 95% CI − 1.02 to 0.55, *I*^2^ = 85%, Fig. 8a). For the FKA assessment, three studies [19, 20, 24] with 269 patients were included in the meta-analysis. The pooled result was 0.71(95% CI 0.05–1.36, *I*^2^ = 0%, Fig. 8b), which means that the FKA of the KA group was significantly greater than that of the MA group. Nine studies [19, 20, 24, 26, 28, 29, 31, 33, 35] compared the mLDFA. The pooled results indicated no significant difference between the two groups (MD = − 0.93, 95% CI − 2.23–0.36, *I*^2^ = 95%, Fig. 8c). Similarly, the difference in FFA (MD = 0.88, 95% CI − 0.23 to 1.99, *I*^2^ = 95%, Fig. 8f) and TS (MD = 0.48, 95% CI − 0.75 to 1.71, *I*^2^ = 87%, Fig. 8g) did not reach statistical significance. However, the pooled MD of nine trials [20, 24, 26, 28, 29, 31–33, 35] with 787 participants indicated that the mMPTA of the two technique groups were different (MD = − 2.64, 95% CI − 3.33 to − 1.95, *I*^2^ = 85%, Fig. 8d). In addition, the pooled result in JLOA was − 2.26 (95% CI − 2.99 to − 1.53, *I*^2^ = 51%, Fig. 8e), suggesting that the JLOA was significantly smaller in the KA group (Fig. 8).

Then, sensitivity analysis was performed when the heterogeneity was higher than 50%. As shown in Additional file 2: Fig. S2, the pooled analysis results of JLOA, FFA, and TS were stable with low heterogeneity. In addition, we found that the heterogeneity of HKA, mLDFA, and mMPTA remained higher than 50% after removing each included study (Tables 4, 5, 6). The robustness of these pooled results was indicated.

**Table 4:** The sensitivity analysis results of HKA

Study excluded	Remaining samples (KA/MA)	Overall effect			Heterogeneity	
		MD	95% CI	*p* value	*I*^2^ (%)	*p* value
Calliess [33]	366/361	0.02	− 0.60 to 0.63	0.95	70	0.0004
Dossett [19]	425/420	− 0.29	− 1.15 to 0.56	0.50	87	< 0.00001
Dossett [20]	422/417	− 0.28	− 1.14 to 0.57	0.52	87	< 0.00001
Kaneda [35]	458/456	− 0.18	− 0.98 to 0.62	0.66	87	< 0.00001
Laende [32]	442/438	− 0.24	− 1.09 to 0.60	0.58	87	< 0.00001
MacDessi [31]	396/393	− 0.31	− 1.18 to 0.56	0.48	86	< 0.00001
Matsumoto [30]	436/431	− 0.46	− 1.11 to 0.20	0.18	75	< 0.0001
Matsumoto [29]	436/431	− 0.13	− 0.96 to 0.71	0.76	86	< 0.00001
McEwen [28]	426/421	− 0.18	− 1.04 to 0.68	0.68	87	< 0.00001
Yeo [26]	436/431	− 0.22	− 1.04 to 0.68	0.62	87	< 0.00001
Young [24]	417/411	− 0.30	− 1.15 to 0.56	0.50	87	< 0.00001

*MD* mean difference

**Table 5:** The sensitivity analysis results of mLDFA

Study excluded	Remaining samples (KA/MA)	Overall effect			Heterogeneity	
		MD	95% CI	*p* value	*I*^2^ (%)	*p* value
Calliess [33]	313/309	− 0.93	− 2.23 to 0.36	0.16	95	< 0.00001
Dossett [19]	372/368	− 0.73	− 2.12 to 0.67	0.31	96	< 0.00001
Dossett [20]	369/365	− 0.77	− 2.18 to 0.65	0.29	96	< 0.00001
Kaneda [35]	405/404	− 0.86	− 2.24 to 0.53	0.23	96	< 0.00001
MacDessi [31]	343/341	− 0.87	− 2.43 to 0.68	0.27	96	< 0.00001
Matsumoto [29]	383/379	− 1.30	− 2.43 to − 0.01	0.05	94	< 0.00001
McEwen [28]	372/368	− 1.35	− 2.44 to − 0.26	0.02	91	< 0.00001
Yeo [26]	383/379	− 0.75	− 2.16 to − 0.67	0.30	95	< 0.00001
Young [24]	364/359	− 0.85	− 2.16 to 0.61	0.25	96	< 0.00001

*MD* mean difference

**Table 6:** The sensitivity analysis results of mMPTA

Study excluded	Remaining samples (KA/MA)	Overall effect			Heterogeneity	
		MD	95% CI	*p* value	*I*^2^ (%)	*p* value
Calliess [33]	296/291	− 2.64	− 3.33 to − 1.95	< 0.00001	85	< 0.00001
Dossett [20]	352/347	− 2.72	− 3.49 to − 1.94	< 0.00001	87	< 0.00001
Kaneda [35]	388/386	− 2.29	− 2.82 to − 1.77	< 0.00001	75	0.0005
Laende [32]	372/368	− 2.67	− 3.44 to − 1.91	< 0.00001	87	< 0.00001
MacDessi [31]	326/323	− 2.85	− 3.51 to − 2.20	< 0.00001	80	< 0.0001
Matsumoto [29]	366/361	− 2.55	− 3.31 to − 1.80	< 0.00001	85	< 0.00001
McEwen [28]	355/350	− 2.77	− 3.65 to − 1.88	< 0.00001	87	< 0.00001
Yeo [26]	366/361	− 2.68	− 3.50 to − 1.86	< 0.00001	87	< 0.00001
Young [24]	347/341	− 2.70	− 3.48 to − 1.93	< 0.00001	87	< 0.00001

*MD* mean difference

### Perioperative results

**Fig. 9:**
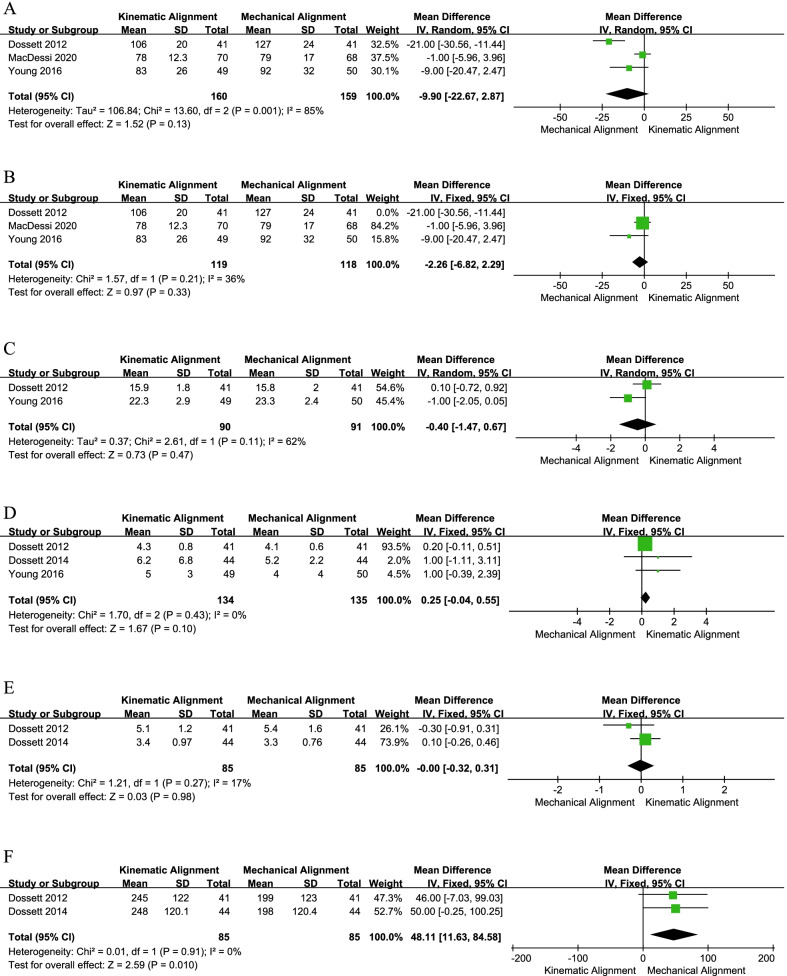
Forest plot of perioperative results between kinematic alignment and mechanical alignment in total knee arthroplasty. **a** OT, **b** the sensitivity analysis results of OT, **c** WL, **d** HS, **e** CHb, **f** WD. *OT* operative time, *WL* wound length, *HS* hospital stay, *CHb* change in hemoglobin, *WD* walking distance

The pooled results of three articles [19, 24, 31] showed that the KA group had a similar operation time compared with the MA group (MD = − 9.90, 95% CI − 22.67 to 2.87, *I*^2^ = 85%, Fig. 9a). When one study [19] was excluded from the meta-analysis, the *I*^2^ dropped to 36% and the result was consistent with the previous pooled result (MD = − 2.26, 95% CI − 6.82 to 2.29, *I*^2^ = 36%, Fig. 9b), indicating the stability of this meta-analysis. Simultaneously, there was no significant difference between the KA and MA techniques in terms of WL (MD = − 0.40, 95% CI − 1.47 to 0.67, *I*^2^ = 62%, Fig. 9c), HS (MD = 0.25, 95% CI − 0.04 to 0.55, *I*^2^ = 0%, Fig. 9d), or CHb (MD = − 0.00, 95% CI − 0.32 to 0.31, *I*^2^ = 17%, Fig. 9e). However, two trials [19, 20] compared the WD, and the mean WD for the KA technique group was significantly longer than that of the MA technique group (MD = 48.11, 95% CI 11.63–84.58, *I*^2^ = 0%, Fig. 9f).

### Complications

**Fig. 10:**
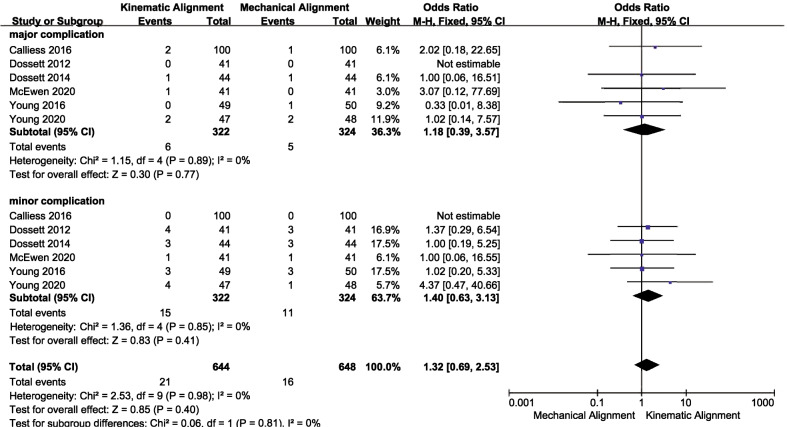
Forest plot of complications rate between kinematic alignment and mechanical alignment in total knee arthroplasty. *M–H* Mantel–Haenszel test

Six studies [19, 20, 24, 25, 28, 33] provided the proportion of participants who experienced complications after the operation. As presented in Fig. 10, there were no significant differences between the two groups (KA:21/322, MA: 16/324, OR 1.32, 95% CI 0.69–2.53, *I*^2^ = 0%). In parallel, the pooled results also revealed that these two techniques had similar outcomes in terms of minor complications (KA: 15/322, MA: 11/324, OR 1.40, 95% CI 0.63–3.13, *I*^2^ = 0%) and major complications (KA: 6/322, MA: 5/324, OR 1.18, 95% CI 0.39–3.57, *I*^2^ = 0%).

## Discussion

The accurate alignment of the lower limb is one of the essential elements influencing the postoperative outcomes and prosthesis survival in patients undergoing TKA. Currently, KA and MA are two primary alignment methods used in TKA. While some RCTs and meta-analyses compared clinical data on the outcomes of KA and MA, the optimal knee alignment for TKA has been inconclusive. Moreover, several new RCT [25, 29, 35] have been published from the previous meta-analysis. Hence, we conducted this updated meta-analysis, aiming to further compare the outcomes of these two alignment methods for TKA. The major finding of this study was that the KA group achieved a better functional outcome than the MA group. Compared with the MA group, the KA techniques with better KSS (knee), KSS (combined), and WOMAC scores also had better knee flexion results. In terms of radiological results, the KA technique resulted in a slightly greater FKA, and the implant alignment was slightly more varus in the tibia than with MA. In addition, the JLOAs in the KA groups were smaller than those in the MA group. Regarding the perioperative results, we identified that the KA group showed a longer walk distance than the MA group. However, there were no significant differences in other knee function parameters, radiological outcomes, perioperative results, or complication rates between these two groups.

A significant number of patients were dissatisfied after TKA with the traditional MA technique [36]. With the development of new technologies, KA has been widely applied in TKA, and the functional results of the KA group seem to be better than those of MA [37]. In this updated meta-analysis, we found better knee function results in the KA techniques in KSS (knee), KSS (combined), and WOMAC scores. These results are similar to those of several previous studies. For instance, a previous meta-analysis that included 529 participants compared the WOMAC score between the two groups and indicated that the KA groups acquired a better outcome score [22]. Similarly, Gao et al. combined 11 papers in a meta-analysis and revealed that the KA technique resulted in a better KSS than MA [21]. In a recent RCT, Matsumoto et al. compared 60 patients who underwent TKA and indicated that the KA group could perform the functional activities in mentioned in the KSS better [29]. The reasons for functional outcome improvement in the KA group may be attributed to the restoration of the knee to its pre-arthritis state, as much as possible. It usually requires less loosening of the ligaments and soft tissues [19], which helps preserve the surrounding soft tissues and the original knee joint line [38]. In addition, our updated meta-analysis showed that the KA group was associated with a greater flexion ROM than the MA group, which was consistent with the results in some previous studies [21, 39, 40]. For instance, Gao et al. compared 287 participants in the KA technique group and 287 participants in the MA technique group and found that the KA group had a higher ROM of flexion [21]. However, another study by Luo et al. [22] demonstrated no significant difference in the ROM between the two techniques. This may be due to differences in the data acquisition and analysis. Since we only pooled the data from RCTs, while studies including RCTs, prospective cohort studies (PCSs), and retrospective cohort studies (RCSs) were all enrolled in their study.

Despite the clinical advantages of the KA technique, it also has some shortcomings of KA for TKA. Our updated meta-analysis implicated a remarkable difference between the KA and MA groups in mMPTA but not in mLDFA. This indicated that the tibia component of KA was more varus than that of MA. A consistent result was obtained in a previous meta-analysis performed by Luo et al., where the mMPTA differed between the two groups [22]. Meanwhile, many studies have shown that the increase in varus tilt in the knee after KA can significantly increase contact stresses and wear of the knee compartment, which will cause implant loosening [41–43]. In contrast, another study indicated that the implant survival rate of the prosthesis remained at an adequate level between the two groups [44]. Moreover, our meta-analysis and previous meta-analysis showed that the rates of complications were similar between the two groups [21, 22, 39]. Thus, the increased risk of surgical failure for KA in TKA may not last. However, this follow-up time is still relatively short, and it is essential to perform a longer follow-up study to elucidate implant survival with KA [28]. In terms of perioperative results, we found that the KA technique had a longer walk distance before discharge than the MA technique [22]. This may also explain why patients with KA for TKA may have better satisfaction than those with MA. However, the perioperative outcomes might be affected by some uncontrollable factors, including the surgical scheme and surgical skill.

Previous studies have shown that it is challenging to avoid heterogeneity in meta-analyses, which may affect the stability of the analysis results [45]. Therefore, we performed a sensitivity analysis to assess whether any individual study would affect the pooled results. By dropping each study and recalculating the combined estimate on the remaining analyses, we found that the most combined results were consistent and without apparent fluctuation. For instance, after omitting the study performed by Dosset et al. [20], the *I*^2^ of ROM (flexion) from dropped 51% to 31%, and the pooled result demonstrated that the KA technique still had a higher ROM of flexion than MA. Hence, these sensitivity analysis results further confirmed the stability of our results.

Despite an effort to make a comprehensive analysis, some inherent limitations of this study should be addressed. First, although we performed a sensitivity analysis, there was still significant heterogeneity in some outcomes, such as HKA. Previous meta-analyses also have this problem [21, 46], which may be attributed to the different surgical techniques, prosthesis types, rehabilitation training, and genetic heterogeneity of the population [46]. Second, the present study only retrieved English articles, which might bias the analysis results to some extent. Third, the follow-up time of the included studies varied, including mainly short, and medium-term RCTs, and there were few studies with long-term follow-up clinical outcomes. Finally, although we have enrolled the latest RCTs, the number of eligible studies and sample size were still small. Thus, a comparable long-term follow-up time, large sample size, and high-quality RCTs will be needed to further our results.

## Conclusion

In conclusion, our study showed that KA in TKA had better functional results than MA in terms of WOMAC scores and KSS (knee and combined). However, KA and MA TKA achieved similar radiological parameters and complication rates.

## Supplementary Information

**Additional file 1: Fig. S1**. The funnel plot for the symmetrical may indicate a low publication bias.

**Additional file 2: Fig. S2**. The sensitivity analysis results of JLOA, FFA, and TS. A. JLOA, B. FFA, C. TS.

## Data Availability

All data and material generated or analyzed during this study are included in this published article.

## Abbreviations

OA: Osteoarthritis
TKA: Total knee arthroplasty
KA: Kinematic alignment
MA: Mechanical alignment
HKA: Hip–knee–ankle angle
RCTs: Randomized control trials
KSS: Knee Society Score
KOOS: Knee Injury and Osteoarthritis Outcome Score
WOMAC: Western Ontario and McMaster Universities Osteoarthritis Index
OKS: Oxford Knee Score
EQ-5D: EuroQoL 5-dimension questionnaire
FJS: Forgotten Joint Score
ROM: Range of motion
FKA: Femoral knee angle
mMPTA: Mechanical medial proximal tibial angle
mLDFA: Mechanical lateral distal femoral angle
JLOA: Joint line orientation angle
TS: Tibial slope
FFA: Femoral flexion–extension angle
OT: Operative time
CHb: Change in hemoglobin
WL: Wound length
WD: Walking distance
HS: Hospital stay
MD: Mean difference
CI: Confidence interval
OR: Odds ratio
