# Supplementary figures and images for: Kinematic alignment versus mechanical alignment in primary total knee arthroplasty: an updated meta-analysis of randomized controlled trials
Source: J Orthop Surg Res. 2022 Apr 4;17:201. doi: 10.1186/s13018-022-03097-2 (PMC8981671; doi:10.1186/s13018-022-03097-2)

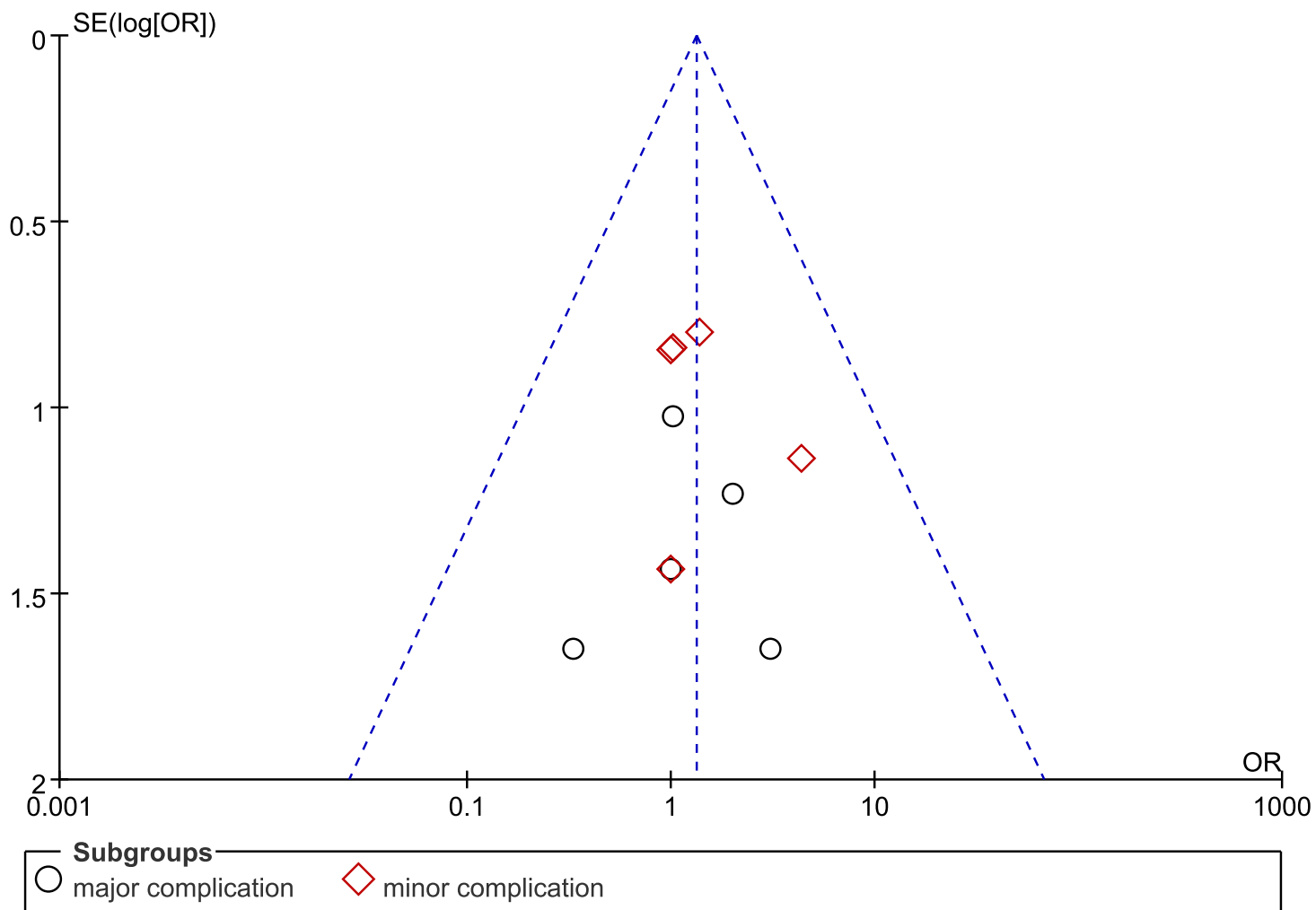

Supplement: Supplementary file 1 — Additional file 1: Fig. S1. The funnel plot for the symmetrical may indicate a low publication bias. [file 13018_2022_3097_MOESM1_ESM.pdf]

A

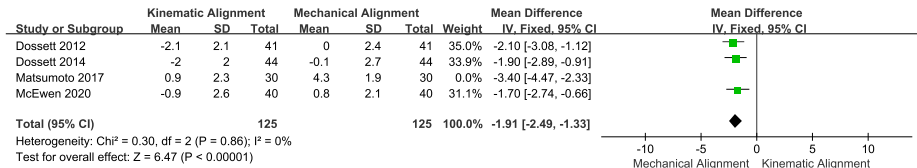

B

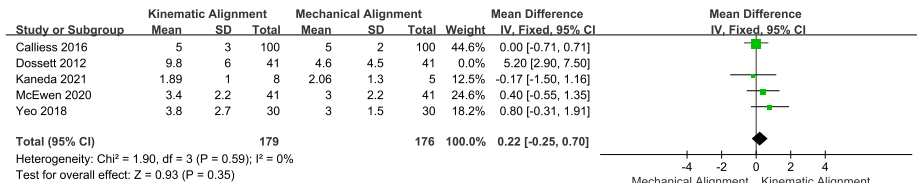

C

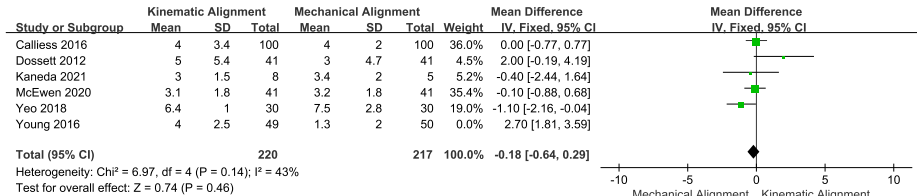

Supplement: Supplementary file 2 — Additional file 2: Fig. S2. The sensitivity analysis results of JLOA, FFA, and TS. A. JLOA, B. FFA, C. TS. [file 13018_2022_3097_MOESM2_ESM.pdf]
